# An Observational Study of the First Experience with Bevacizumab for the Treatment of Patients with Recurrent High-Grade Glioma in Two Belgian University Hospitals

**DOI:** 10.1155/2012/801306

**Published:** 2012-03-13

**Authors:** M. Huylebrouck, S. Lv, J. Duerinck, A. Van Binst, I. Salmon, J. De Greve, O. De Witte, S. Luce, A. Michotte, J. D'Haens, B. Neyns

**Affiliations:** ^1^Department and Laboratory of Medical Oncology, UZ Brussel, Laarbeeklaan 101, 1090 Brussels, Belgium; ^2^Department of Neurosurgery, UZ Brussel, Laarbeeklaan 101, 1090 Brussels, Belgium; ^3^Department of Radiology, UZ Brussel, Laarbeeklaan 101, 1090 Brussels, Belgium; ^4^Department of Pathology, ULB Hôpital Erasme, Route de Lennik 808, 1070 Brussels, Belgium; ^5^Department of Neurosurgery, ULB Hôpital Erasme, Route de Lennik 808, 1070 Brussels, Belgium; ^6^Department of Medical Oncology, ULB Hôpital Erasme, Route de Lennik 808, 1070 Brussels, Belgium; ^7^Department of Pathology and Neurology, UZ Brussel, Laarbeeklaan 101, 1090 Brussels, Belgium

## Abstract

*Background*. Bevacizumab (BEV), a humanized immunoglobulin G1 monoclonal antibody that inhibits VEGF has demonstrated activity against recurrent high-grade gliomas (HGG) in phase II clinical trials. *Patients and Methods*. Data were collected from patients with recurrent HGG who initiated treatment with BEV outside a clinical trial protocol at two Belgian university hospitals. *Results*. 19 patients (11 M/8 F) were administered a total of 138 cycles of BEV (median 4, range 1–31). Tumor response assessment by MRI was available for 15 patients; 2 complete responses and 3 partial responses for an objective response rate of 26% for the intent to treat population were observed on gadolinium-enhanced T1-weighted images; significant regressions on T2/FLAIR were documented in 10 out of 15 patients (67%). A reduced uptake on PET was documented in 3 out of 4 evaluable patients. The six-month progression-free survival was 21% (95% CI 2.7–39.5). Two patients had an ongoing tumor response and remained free from progression after 12 months of BEV treatment. *Conclusions*. The activity and tolerability of BEV were comparable to results from previous prospective phase II trials. Reduced uptake on PET suggests a metabolic response in addition to an antiangiogenic effect in some cases with favorable clinical outcome.

## 1. Introduction

Gliomas are the most frequent primary tumors of the central nervous system (CNS) and represent approximately 2% of all malignant diseases. Their annual incidence is about 11.5 new cases per 100.000 persons per year [1, 2]. The WHO classification of tumors of the central nervous system distinguishes the subtypes of glioma according to morphology and grade [3]. High-grade gliomas (HGG, WHO-grade 3 and 4 glioma) are malignant tumors with a poor survival outcome. In a pivotal phase III trial, where patients diagnosed with glioblastoma (GB, WHO-grade IV glioma) who were treated with postoperative radiation therapy (RT) and concomitant temozolomide (TMZ) followed by six cycles of adjuvant TMZ, the median survival was 14,6 months, while the overall survival (OS) was 27.2% at 2 years, 16.0% at 3 years, 12.1% at 4 years, and 9.8% at 5 years [4]. The prognosis of patients with WHO-grade III glioma is superior to that of GB patients but much more heterogeneous and correlated with the histopathological and molecular-genetic subtype [5, 6]. Following initial resection and postoperative RT, anaplastic gliomas recur after a median of 2-3 years. Most often, recurrent grade III glioma will have transformed into a more aggressive tumor at recurrence (a so-called *secondary glioblastoma*). The survival of patients with recurrent high-grade glioma following prior therapy with alkylating chemotherapy is grim and no treatment has demonstrated to improve the survival in a randomized clinical trial [6, 7].

HGG are among the most angiogenic tumors and typically express high amounts of vascular endothelial growth factor (VEGF). VEGF is a key molecular mediator of tumor-associated neoangiogenesis and its expression level has been correlated with tumor vascularisation, WHO-grade and prognosis [8, 9]. HGG also express the VEGF-receptors and frequently carry an amplicon of chromosome 4q12 comprising the VEGF-receptor-2 (VEGFR2), PDGFR-alfa, and cKIT genes in 23–30% of cases [10–12]. Coexpression and/or amplification of both the VEGF and VEGFR2 constitute an autocrine/paracrine loop.

Bevacizumab (BEV; Avastin, Roche, Basel, Switzerland) is a humanized immunoglobulin G1 monoclonal antibody that binds to and inhibits VEGF. It has proven to be active in combination with cytotoxic agents and is registered by the FDA and EMA as part of a combination treatment regimen with chemotherapy for metastatic colorectal cancer, non-small-cell lung cancer, and breast cancer, and in combination with interferon-alfa in metastatic renal cell carcinoma [8, 13–19].

On May 5, 2009, the FDA granted accelerated approval to bevacizumab as a single agent for the treatment of patients with recurrent glioblastoma. The approval was based on the results of two phase II clinical trials (AVF3708g and NCI 06-C-0064E) [20]. The largest phase II trial, involving a total of 167 patients with recurrent GB, randomized patients between treatment with BEV (at a biweekly dose of 10 mg/kg) in one arm and the combination of BEV (at the same dose) and irinotecan in a second arm [20]. In the bevacizumab-alone and the bevacizumab-plus-irinotecan groups, the objective response rates were 28.2% and 37.8%, and the estimated 6-month progression-free survival rates were 42.6% and 50.3%, respectively. The number of adverse events in the BEV plus irinotecan population was higher (65.8% versus 46.4% grade ≥3 adverse events), while median overall survival times was comparable between the two arms (9.2 months and 8.7 months, resp., compared to 7.5 months in a historical population [7]).

In a single-arm phase II trial, investigating the sequential use of BEV and the combination of BEV and irinotecan, no activity was found for the combination after failure of BEV as a single agent [21]. BEV has also demonstrated antitumor activity as a single agent in patients with recurrent anaplastic glioma [22, 23]; in combination with a variety of cytotoxic agents [24–26], and when administered on a once every 3-week schedule (at a dose of 15 mg/kg every 3 weeks) [27].

MRI-based tumor evaluation in patients treated for recurrent glioma have been characterized by a rapid regression of tumor-associated edema and restoration of the blood-brain/tumor barrier. Progression of disease by diffuse, non-gadolinium-enhancing infiltration of the brain (=gliomatosis) may occur in patients that respond to BEV [28]. Notwithstanding these atypical patterns of progression, updated overall survival of the patients treated in the BRAIN (AVF3708g) study indicated that 16% of patients remained alive at 30 months of followup, a percentage that compares favorably with historical controls [29]. Besides the effect of BEV on glioma-associated vasculature, responses documented by positron emission tomography (PET) using fluorothymidine (FLT), an imaging marker of cell proliferation, were correlated with an improved overall survival in patients treated with irinotecan and BEV [30, 31].

On 19 November 2009, the CHMP (EMA) refused to change the terms of the marketing authorization for bevacizumab in the EU to include recurrent glioblastoma. From April to November 2009, the Belgian RIZIV/INAMI provided partial (60%) reimbursement for bevacizumab following an individual request to the “bijzonder Solidariteitsfonds/Fonds Spécial de Solidarité.” This paper reports the first experience with bevacizumab for recurrent glioma in patients treated at two Brussels university hospitals.

## 2. Patients and Methods

### 2.1. Data Collection

This observational (noninterventional) study was performed with the clinical data that were retrospectively retrieved from the medical files of all patients with recurrent high-grade glioma who initiated BEV treatment between 9 January 2009 and 27 January 2010. These patients represent the first patients treated with BEV for recurrent glioma at two Belgian University hospitals, the UZ Brussel and ULB Erasme.

We collected data regarding the general clinical and neurological evolution during BEV treatment, the BEV treatment disposition (Table 3), as well as laboratory tests performed during BEV therapy. Adverse events were classified according to the Common Terminology Criteria for Adverse Events v3.0 (CTCAE). Tumor response assessment on MRI was based on the Macdonald criteria [32]. In accordance, we made the sums of the maximal cross-sectional radii of the contrast enhancing tumor measured by consecutive contrast MR. A complete response (CR) was defined as a disappearance of all contrast enhancing tumor, with the patient neurologically improved or stable and off corticosteroids. A partial response (PR) was defined as a 50% or more decrease in the size of the contrast-enhancing tumor with the patient neurologically improved or stable and with the corticosteroid dose stable or decreased. Progressive disease (PD) was defined as a 25% or more increase in the size of the contrast enhancing tumor or appearance of a separate tumor. Stable disease (SD) was defined for all other situations. In addition, we assessed the abnormalities on sequential T2 and FLAIR MRI—sequences in a similar fashion, and changes on PET—scan of the brain for the subgroup of patients evaluated by this imaging modality.

### 2.2. Statistical Analysis

BEV treatment disposition, BEV-related adverse events, demographic and baseline patient, and disease characteristics were summarized using descriptive statistics. Kaplan-Meier statistics were used to estimate the probability of survival (SPSS Inc., Chicago, Illinois 60606, USA).

## 3. Results

### 3.1. Baseline Patient Characteristics

Nineteen patients (11 men and 8 women) with recurrent supratentorial HGG were identified to have received treatment with BEV for progressive disease following failure after prior treatment including surgery, radiation therapy, and chemotherapy. Baseline patient characteristics are summarized in Table 1. The median patient age at the initiation of BEV treatment was 40 years (range 28 to 70). Eighty-nine percent of the patients were younger than 50 years at the start of BEV treatment. Eight (53%) patients had a pathological diagnosis of *primary* GB, 4 (21%) of *secondary* GB, and 5 (26%) of recurrent grade III glioma. The median baseline Karnofsky performance score (KPS) was 70. Five (26%) patients had a baseline KPS of 60% or lower. In six patients, initial treatment consisted of surgery followed by radiotherapy. In 13 patients, the initial treatment consisted of surgery, followed by RT with concomitant TMZ and adjuvant TMZ (median number of adjuvant TMZ cycles: 6, range 1 to 12). Six of these patients experienced progression during adjuvant TMZ treatment. Three patients had been treated with radiation therapy at recurrence (one patient was administered fractionated radiotherapy at a dose of 55,5 Gy, two patients were treated using *γ*-knife radiosurgery). Twelve patients underwent additional modalities of salvage therapy for recurrent disease (chemotherapy, dendritic cell vaccination) before initiating BEV therapy.

### 3.2. Bevacizumab Treatment Disposition

Three patients initiated BEV at a dose of 5 mg/kg every 2 weeks (in one of them, the dose was escalated to 10 mg/kg every 2 weeks after the first administration), 14 patients at a dose of 10 mg/kg every 2 weeks, and 2 patients at a dose of 15 mg/kg every 3 weeks, according to BEV administration regimens published in the literature [20, 21, 24, 27, 33–35].

A total number of 123 BEV treatment cycles were analyzed in this study. Treatment was ongoing in 2 patients at the time of this analysis. A median number of 4 cycles were administered per individual patient (range 1 to 16). There were no dose reductions of BEV. Three patients were simultaneously treated with a cytotoxic drug (hydroxyurea, TMZ, or CCNU). Fourteen patients (74%) were treated with corticosteroids at the initiation of BEV treatment. The dose of corticosteroids could be tapered in 4 patients and stopped in two of them.

### 3.3. Treatment-related Adverse Events

Nine BEV-related adverse events were encountered, of which none were grade 4 or 5 (Table 2). Two grade 3 adverse events (ulceration of skin striae and an abdominal pain syndrome) necessitated stopping BEV administration in the absence of documented tumor progression.

### 3.4. Antitumor Activity and Survival

Four patients (21%) experienced a rapid increase in disease-related symptoms after the initiation of BEV. Their clinical condition prohibited an objective tumor evaluation with MRI after the initiation of BEV. In all 4 patients, clinical deterioration was considered related to progression of disease.

**Figure 1:**
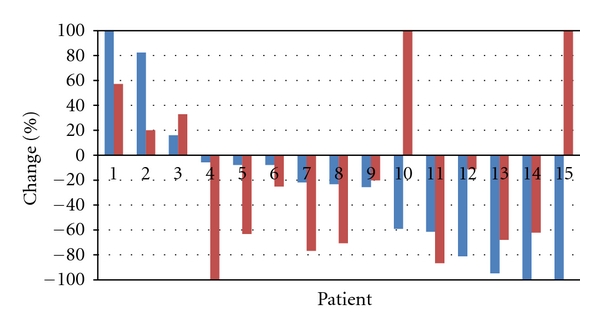
Maximal % change in tumor surface area on gadolinium-enhanced T1-weighted MRI (Blue bar) and surface area of nonenhancing lesions on T2-weighted MRI (Red bar) as compared to baseline during BEV therapy. Patient no. 14 had a decrease of the measured contrast-enhancing lesion; however, a new contrast-enhancing lesion appeared in a different location.

**Figure 2:**
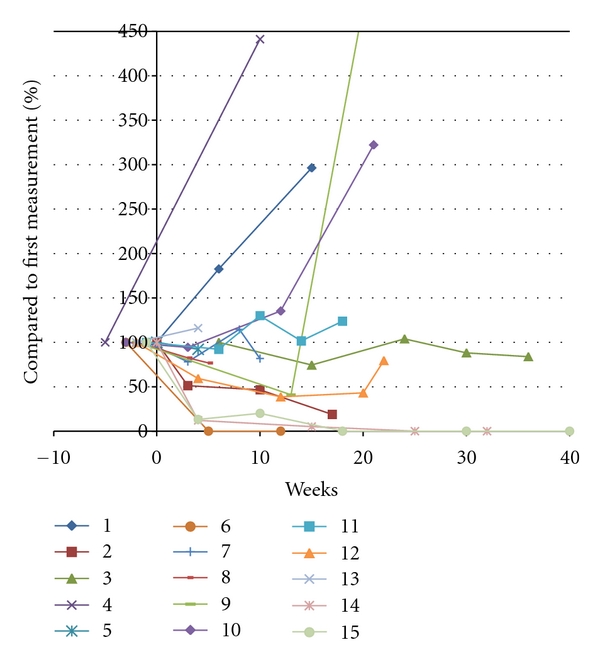
Change in tumor surface area in time during BEV treatment in 15 patients evaluable on gadolinium-enhanced T1-weighted MRI. Patient no. 14 had a decrease of the measured contrast-enhancing lesion; however, a new contrast-enhancing lesion appeared in a different location.

Fifteen (79%) patients were assessable for tumor response on T1 gadolinium-enhanced MRI-sequences (Gd-T1). Tumor regression was complete in 2 patients and more than 50% in an additional 3 patients. This correlates to an objective response rate of 33% according to the MacDonald criteria for the 15 evaluable pts on MRI and 26% for the intent to treat population. All of these patients had stabilization or improvement of disease-related symptoms and none of them had an increase in corticosteroid dose. Seven patients (47%) obtained a stable disease, and 3 (20%) patients experienced immediate progression of disease during BEV therapy (Figures 1 and 2).

**Figure 3:**
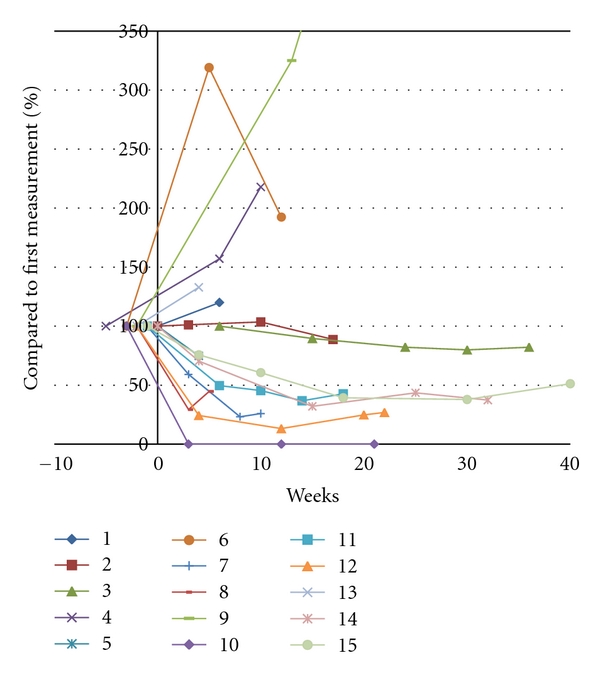
Change in the surface area of nonenhancing lesions in time during BEV treatment in 14 patients evaluable on T2-weighted MRI.

Assessment of tumor response by T2/FLAIR MRI-sequences was available for the same 15 patients who were evaluable by T1-weighted MRI. Complete disappearance of nonenhancing lesions was observed in 1 patient, and partial regression was observed in 6 additional patients (47%; for an objective tumor response according to the RANO criteria of 37%). No change was observed in four patients (27%), and an increase of abnormalities at the first evaluation was observed in 4 patients (27%) (Figures 1 and 3).

**Figure 4:**
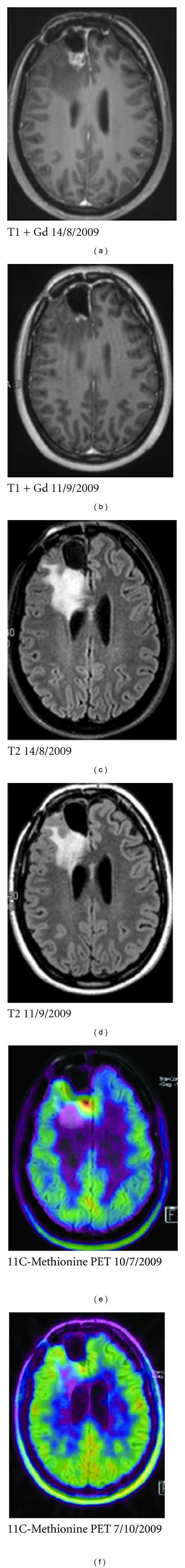
Case illustration of a tumor response on BEV therapy. Baseline images by gadolinium-enhanced T1 MRI, T2, and 11C-Methionine-PET on the left hand (top to bottom) and images obtained after 2 administrations of BEV. The patient remained free from progression after more than 1 year of BEV therapy.

Four patients were evaluated by 2-(18F)-Fluoromethyl-L-phenylalanine PET (FMP-PET) or 11C-Methionine-PET of the brain before and during BEV treatment. A reduced uptake of amino-acid tracer on PET-scan was documented in 3 out of 4 pts during BEV treatment, in 2 patients with the most favorable progression-free survival, and a complete normalization of PET-tracer uptake was observed during BEV therapy (Figure 4).

As of October 2010 (the time of this analysis), 16 patients had died, all disease related. One patient was lost to follow-up after progression on BEV therapy. Two patients (10,5%) remained free-from progression after 1 year of BEV treatment. In one of these patients BEV was stopped after 1-year of therapy in the absence of metabolic activity on methionine-PET and normalization of gadolinium enhancement on T1-MRI. Three months after stopping BEV, the patient developed progression of disease. The second patient developed progression of disease following 18 months of remission on BEV therapy.

**Figure 5:**
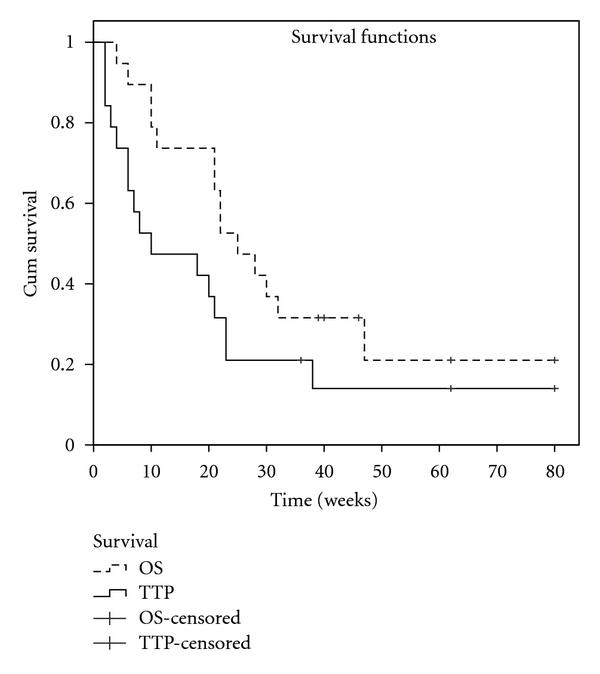
Kaplan-Meier progression-free survival and overall survival estimates. OS—overall survival, TTP—time to progression. The 6mPFS% was 21% (95% CI 2.7–39.5); the 6mOS% was 47.4% (95% CI 24.8–69.9). The median PFS was 10 weeks (95% CI 2–25); the median OS was 25 weeks (95% CI 17–32).

The six-month progression-free survival rate (6mPFS%) was 21% (95% CI 2.7–39.5), and the 6mOS% was 47.4% (95% CI 24.8–69.9). The median PFS and overall survival (OS) were 10 weeks (95% CI 2–25) and 25 weeks (95% CI 17–32), respectively (Figure 5).

## 4. Discussion

High-grade gliomas are highly aggressive and therapy resistant malignant tumors. With contemporary standard treatment options for patients diagnosed with GB, the prognosis remains grim and most patients do not survive for more than 2 years following the diagnosis [4]. Salvage therapies with cytotoxic agents are seldom successful (<10% ORR) [7, 36]. Uncontrolled clinical studies with the VEGF targeted IgG1 monoclonal antibody bevacizumab have shown unprecedented tumor response rates and survival outcomes that compare favorably with historical control series. Within the context of these prospective clinical trials, BEV-associated toxicities have been acceptable and reflect a typical spectrum of side effects that are associated with VEGF(R) targeted therapies. As most of the prospective trials have used quite stringent patient recruitment criteria, safety and activity of BEV when used outside of a clinical trial merit consideration.

We, therefore, retrospectively analyzed the clinical outcome of nineteen patients treated with BEV for recurrent HGG during the first year that BEV became available in this indication at two university hospitals in Belgium. As expected, the baseline characteristics of the patients included in our analysis compared unfavorably with those of the patients treated in the pivotal phase II trial [20]. A larger proportion of patients treated in our series were treated at second or third relapse, and the baseline KPS was less or equal to 60% in a significant proportion of patients. Nevertheless, BEV therapy was generally well tolerated. We, therefore, consider that the results from our study, although preliminary, indicate that the safety profile of BEV for recurrent HGG outside the context of a prospective clinical trial is comparable to what has been reported in the literature. Nevertheless, two patients needed to stop treatment because of BEV-related side effects in the absence of documented progression. It, therefore, needs to be considered that frail patients might be at higher risk for BEV-related adverse events. The objective tumor response, either analyzed by gadolinium-enhanced T1 MRI and/or T2/FLAIR imaging, was interestingly high in our patient population. Reflecting the poor baseline prognostic characteristics of our population, both time to progression (TTP) and overall survival (OS), in contrast, were low when compared to published series. However, two patients in our series experienced a durable complete response and progression-free survival for over 1 year following BEV therapy for recurrence, indicating the potential for a durable therapeutic effect in a subgroup of patients.

Patterns of tumor response and progression during antiangiogenic therapy are a matter of controversy in the recent literature. In our small series, the tumor response pattern to BEV was heterogeneous and could be divided in three distinct patterns. A first group of patients demonstrated no evidence of response (clinical or radiological) to bevacizumab therapy. Such was the case in four patients, who deteriorated rapidly and could not be evaluated by MRI, and three (20%) of the fifteen assessable patients on gadolinium-enhanced T1 MRI. A second group of patients initially responded to therapy on gadolinium-enhanced T1 MRI, but subsequently showed early (<6 months) regrowth of the gadolinium-enhancing tumor mass (*n* = 7; 47%) or deteriorated clinically without characteristic increase in the diameter of gadolinium-enhancing T1 MRI abnormities (*n* = 3, 20%). In these 3 patients, there was a marked progression of abnormalities on T2/FLAIR MRI, most likely representing VEGFR-independent tumor cell infiltration of the brain. A small third group of patients (2 patients; 13%) experienced a very favorable and sustained tumor response to BEV therapy, evident on both Gd-T1 and T2/FLAIR MRI. Further useful differentiation of response to BEV may be obtained by metabolic tumor imaging using PET. PET-imaging has proven to be useful in assessing the response of recurrent glioma treated with a variety of modalities. Likewise, FLT-PET has been correlated with clinical outcome of patients treated with the combination of irinotecan and bevacizumab [30]. In our series, normalization in PET tracer accumulation was observed in the 2 cases with the most favorable evolution on MRI and survival. These case observations indicate that single agent BEV can be associated with a reduction of PET-tracer uptake by the tumor, suggestive of a metabolic effect. These observations merit further study of PET as a tool for response assessment in patients with recurrent glioma treated by BEV. PET response may be more predictive for survival as opposed to response assessment by MRI.

We conclude that our analysis of the first experience with BEV for the treatment of patients with recurrent HGG is in line with the reported tolerability and activity of this new treatment from prospective clinical studies. Our observations support the usefulness of BEV as a new treatment option for patients with recurrent HGG taken into account the absence of alternative treatment options with proven activity. Further observational study of the use of BEV in this indication should be considered to optimize its use in daily practice. Correlative studies between clinical, radiological, PET parameters with molecular-genetic features of the HGG should be conducted to provide predictive markers for response and survival benefit from BEV.

## Figures and Tables

**Table 1:** Patient baseline characteristics.

Variable	No. of patients	%
Treated population	19	100
Age of treated population		
Median (range)	40 (28 to 70)	
Sex of treated population		
Male/female	11/8	58/42
Tumor location		
Frontal	9	47
Temporal	5	26
Parietal	3	16
Occipital	1	5
Thalamus	1	5
Initial tumor histology		
Astrocytoma	1	5
Anaplastic astrocytoma	3	16
Anaplastic oligodendroglioma	1	5
Anaplastic oligoastrocytoma	5	26
Glioblastoma	9	47
Latest tumor histology		
Anaplastic astrocytoma	2	10
Anaplastic oligodendroglioma	0	0
Anaplastic oligoastrocytoma	3	16
Glioblastoma	14	74
Surgery at primary diagnosis	18	95
Total resection	6*	32
Subtotal resection	7	37
Biopsy	3*	16
Unknown extent	3	16
Radiotherapy at primary diagnosis	19	100
Concurrent temozolomide	15	79
Adjuvant temozolomide	13	68
Surgery for relapse	10	53
Radiotherapy for relapse	3	16
Fractionated	1	5
*γ*-knife	2	11
Temozolomide for relapse	7	37
Salvage therapy prior to BEV therapy°	12	63
CCNU	3	16
Dendritic cell vaccine	4	21
PCV	2	11
REGAL study: CCNU+cediranib/placebo	5	26
Sutent study: Sunitinib/CCNU	3	16

*One patient had a biopsy, followed by gross tumor resection.

°Seven patients had one salvage therapy prior to BEV therapy.

Five patients had the salvage therapies prior to BEV therapy.

**Table 2:** BEV related adverse events.

Description	Grade 1	Grade 2	Grade 3	Total	Treatment regimen
Likely related to BEV therapy					
Hypertension	1	1	0	2	10 mg/kg/2weeks
Epistaxis	0	1	0	1	10 mg/kg/2weeks
Ulceration skin	1	0	1	2	10 mg/kg/2weeks
Hematochezia	1	0	0	1	1 cycle at 5 mg/kg/2weeks;
					9 cycles at 10 mg/kg/2weeks
Subungual hemorrhage	1	0	0	1	10 mg/kg/2weeks
Wound dehiscence	0	1*	0	1	10 mg/kg/2weeks
Abdominal pain syndrome	0	0	1	1	10 mg/kg/2weeks
Total	4	3	2	9	

*Occurred more than 2 months after termination of BEV therapy.

**Table 3:** BEV treatment disposition.

Variable	Total (median, range)
Number of BEV cycles	123 (4, 1–16)
	Number of patients (%)

Patients treated at a dose of 5 mg/kg every 2 weeks	3* (15,8)
Patients treated at a dose of 10 mg/kg every 2 weeks	15* (78,9)
Patients treated at a dose of 15 mg/kg every 3 weeks	2 (10,5)

*One patient had a dose escalation from 5 to 10 mg/kg every 2 weeks after the first cycle.
